# Effects Induced by the Agricultural Application of Probiotics on Antioxidant Potential of Strawberries

**DOI:** 10.3390/plants11060831

**Published:** 2022-03-21

**Authors:** Rima Mockevičiūtė, Sigita Jurkonienė, Virgilija Gavelienė, Elžbieta Jankovska-Bortkevič, Božena Šocik, Gabija Armalytė, Rimas Budrys

**Affiliations:** 1 Nature Research Centre, Institute of Botany, Laboratory of Plant Physiology, Akademijos Street 2, LT-08412 Vilnius, Lithuania; sigita.jurkoniene@gamtc.lt (S.J.); virgilija.gaveliene@gamtc.lt (V.G.); elzbieta.jankovska@gamtc.lt (E.J.-B.); bozena.socik@gamtc.lt (B.Š.); gabija.arm@gmail.com (G.A.); 2 Baltic Probiotics, Bakery, Rucavas Parish, LV-3477 South Kurzeme Region, Latvia; balticprobiotics@gmail.com

**Keywords:** anthocyanins, antioxidant activity, ascorbic acid, *Fragaria ananassa*, phenols, plant probiotic microorganisms

## Abstract

With the recent rapid development of the functional food sector, agriculture is looking for alternatives to improve the quality of food grown by limiting chemical fertilizers. This study evaluated the effects of two commercial plant probiotics, ProbioHumus and NaturGel, on the growth and quality of strawberry fruits. Strawberry plants were sprayed with microbial probiotics twice a year: after harvesting at the beginning of dormancy and at the stage of leaf development. Spray applications of ProbioHumus, NaturGel, and NaturGel + ProbioHumus in the organic farm fields significantly increased the fresh fruit weight up to 42%, 35%, and 37%, respectively, compared to the non-treated control. An increase in the weight of fresh strawberry fruits may be associated with an increase in dry matter accumulation. The probiotics had a positive effect on the total content of phenols, anthocyanins, and especially ascorbic acid in strawberry fruits. The increase in ascorbic acid in strawberry fruits was up to 97% compared to the non-treated control. The fruits from plants inoculated with probiotics showed significantly higher antioxidant activity. In summary, ProbioHumus and NaturGel are effective tools for improving the quality of strawberries and can be exploited in sustainable agriculture as a tool for adding value to functional food.

## 1. Introduction

Strawberries (*Fragaria ananassa* Duch.) belong to the *Rosaceae* family and the genus *Fragaria* and are one of the most important fruit crops grown in the world and Lithuania. The cultivation of strawberries started around 250 years ago [1]. The plant is acclimatized to a variety of environments and can be grown worldwide, including in tropical, subtropical, and temperate climates. Strawberry fruits are valued not only for their sensory properties—sweet taste, attractive aroma, and smooth texture—but also for their extraordinary biological value and potential health benefits, being characterized as a functional food [2,3,4,5,6]. They are a unique source of nutrients and bioactive compounds, being rich in trace elements, B vitamins, vitamin C, vitamin E, folates, carotenoids, and various polyphenols [2,3,6]. The scientific literature provides an increasing number of epidemiological and clinical studies confirming the protective effect of strawberries in reducing obesity, hyperglycemia, hyperlipidemia, hypertension, inflammatory rates, neurodegenerative disorders, cancer mortality, and cardiovascular diseases [2]. The health effects associated with the consumption of strawberry fruits are attributed to their antioxidant properties [6,7,8]. Several studies have shown that strawberries have a high antioxidant capacity, up to 10-fold higher than many other fruits, including oranges, kiwis, grapefruit, persimmons, and apples [9,10], and are similar to berries such as raspberries and blackberries and red grapes [4,7,11]. Sun and coworkers [7] found that 100 g of strawberries contains 1130 mg of vitamin C and has a strong antioxidant effect, being the fourth most active of the 11 fruit extracts tested. Naturally occurring effective antioxidants: ascorbic acid, ellagitannins, and anthocyanins, have been shown to have the greatest effect on the antioxidant capacity of strawberry fruits [12,13]. All the same, the amount of phytochemicals in strawberries and the antioxidant activity of fruit can be significantly affected by differences in varieties and agricultural practices [6,14,15,16].

A number of studies have shown that organically grown strawberries have higher antioxidant enzyme (such as glutathione peroxidase, glutathione reductase, catalase, superoxide dismutase, ascorbate peroxidase, dehydroascorbate reductase, guaiacol peroxidase, monodehydroascorbate reductase) activity, antioxidant capacity, and levels of antioxidants [14,16,17]. Extracts from organically grown strawberries have also been shown to have significantly higher antiproliferative activity on colon HT29 and breast MCF-7 cancer cells [8]. It is clear that agricultural practices that affect the nutritional value of fruit deserve more attention. As a result, there is an increasing focus on organic farming practices, but the problem of declining yields is often encountered here [18,19]. Organic farming systems rely on biofertilization to maintain soil productivity as much as possible [20]. In recent times, one of the most widely used alternative technologies is the use of biofertilizers in combination with microorganisms such as plant probiotics [21,22,23]. Complexes of microorganisms that improve plant nutrition and support plant development by various mechanisms can be defined as plant probiotics [20,21,23,24,25,26,27]. Naturally occurring probiotic bacteria associated with plants stimulate plant growth through various direct and indirect mechanisms, produce a variety of compounds such as plant hormones, antibiotics, and lytic enzymes, fix weather nitrogen, and decompose soil mineral nutrients [24,25,28,29,30]. Several bacterial species have been identified to be useful for plant growth, yield, and crop quality [20], namely, *Bacillus*, *Paraburkholderia*, *Pseudomonas*, *Acinetobacter*, *Alcaligenes*, *Arthrobacter*, and *Serratia*. Some studies have shown that plant probiotic microorganisms promote growth and increased yields in several fruit species, including strawberries. Several papers have reported a beneficial influence of probiotics on the synthesis and production of ascorbic acid and B vitamins [31,32] and an increased content of phenolic compounds (anthocyanins and flavonoids) [23,25] in strawberry fruits. However, there is a lack of studies investigating the effects of plant probiotics on strawberry fruit quality assessment under natural conditions at organic farms.

In recent years, the commercialization of biofertilizers has been growing, and the number of products offered by companies has been expanding [26]; however, the performance of plant probiotic microorganisms at the field scale is not always satisfactory. Therefore, studies of selected plant probiotic microorganisms are important because of their ability to properly nourish crops and ensure long-term soil fertility in open field conditions [33].

The aim of this research was to investigate the effect of the use of plant probiotics on the growth and phytochemical characteristics of strawberries grown on an organic farm. The specific tasks of the survey were to determine the impact of two commercial plant probiotics, ProbioHumus and NaturGel, on strawberry growth and to evaluate the effect of these probiotics on fruit biofunctional properties by total phenol, anthocyanin, and ascorbic acid contents and antioxidant activity.

## 2. Results

### 2.1. The Impact of ProbioHumus and NaturGel on the Strawberry Fruit Biometric Parameters

**Figure 1:**
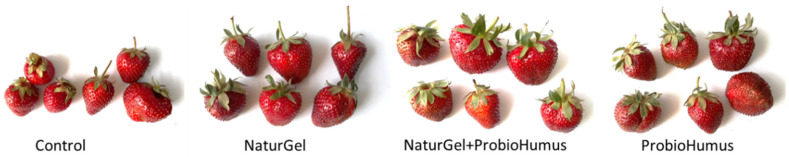
Strawberry (*Fragaria ananassa*) cv. ‘Deluxe’ fruits from plants treated with water (Control), NaturGel (40 mL ha^−1^), NaturGel + ProbioHumus (40 mL ha^−1^ + 40 mL ha^−1^), and ProbioHumus (40 mL ha^−1^) grown on an organic farm.

The probiotic preparations ProbioHumus and NaturGel, either alone or in combination, significantly increased the biomass of fresh and dry strawberry fruits compared to the control group (Table 1). ProbioHumus had the greatest effect on fruit formation, with a 42.4% increase in fresh weight. The fresh mass of the fruits increased by 35.6% and 37.3% with NaturGel and NaturGel in combination with ProbioHumus, respectively, compared to the control (Table 1, Figure 1). The NaturGel treatment resulted in a significant increase in the diameter of the strawberry fruits, while the NaturGel and NaturGel + ProbioHumus treatments resulted in a significant increase in strawberry fruit length. 

### 2.2. The Impact of Probiotics on the Content and Activity of Antioxidants in Strawberry Fruits

#### 2.2.1. Total Phenols

**Figure 2:**
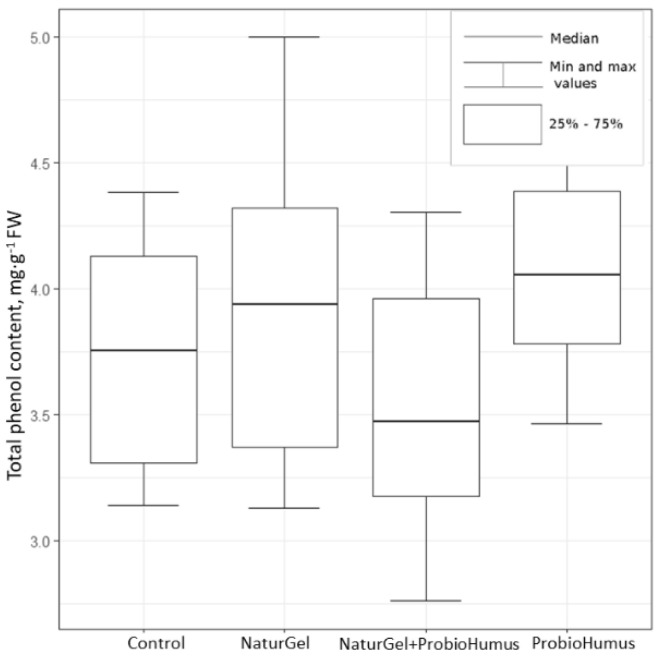
The impact of the probiotic preparations NaturGel and ProbioHumus on the content of phenols (mg GAE g^−1^ FW) in fresh strawberry fruits. The Shapiro–Wilk criterion was used to determine the normality of the data, Levene’s test was used to assess the variance, and the Wilcoxon–Mann–Whitney (WMW) criterion was used to assess the level of significance of the data.

The results of the total phenol content obtained by the Folin–Ciocalteu assay showed that the probiotics did not have a statistically significant effect on the total phenol content of strawberry fruits. Although the median total phenol content in control plants (3.75 mg GAE g^−1^ FW) was lower than the median in strawberry fruits harvested from plants treated with ProbioHumus (4.06 mg GAE g^−1^ FW, Kruskal–Wallis criterion, χ^2^ (1) = 3.35, *p* > 0.05) and NaturGel (3.93 mg GAE g^−1^ FW, Kruskal–Wallis criterion, χ^2^ (1) = 0.40, *p* > 0.05), the increase was not statistically significant due to high scattering (Figure 2).

#### 2.2.2. Total Anthocyanin Content

**Figure 3:**
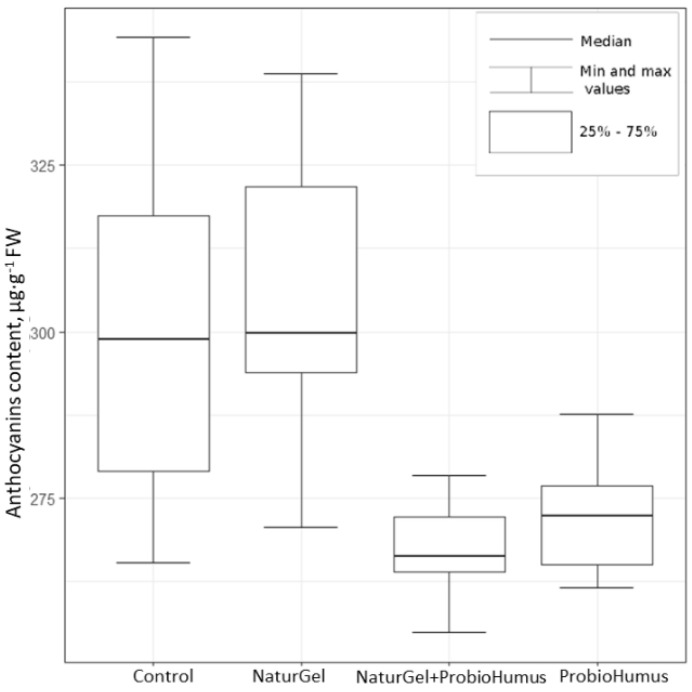
The impact of the probiotic preparations NaturGel and ProboHumus on the content of anthocyanins in fresh strawberry fruits. Statistical analysis criteria are as indicated in Figure 2.

The effect of NaturGel and ProbioHumus on the total anthocyanin amount in strawberries was measured using pH differential analysis. The results show that the total anthocyanin content in the fruits of plants treated with NaturGel reached up to 35.8 mg 100 g^−1^ FW and did not differ from the control. This mean value was the highest among further treatments. The ProbioHumus treatment (Kruskal–Wallis criterion, χ^2^ (1) = 3.87, *p* < 0.05) and the combination NaturGel and ProbioHumus (Kruskal–Wallis criterion, χ^2^ (1) = 0.20, *p* < 0.05) resulted in a lower concentration of anthocyanins in the fruit of strawberries compared to the control (Figure 3).

#### 2.2.3. Ascorbic Acid

**Figure 4:**
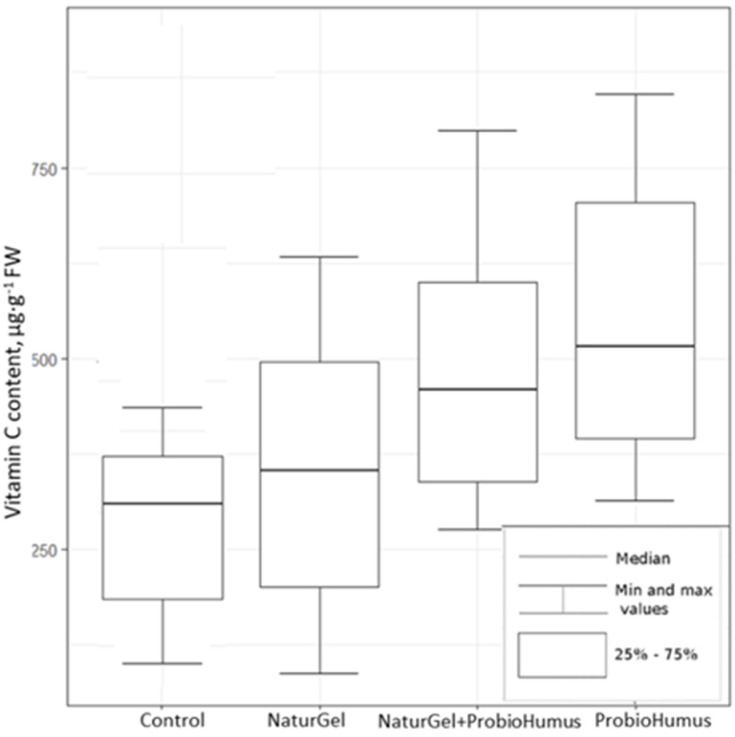
The impact of the probiotic preparations NaturGel and ProbioHumus on the content of ascorbic acid in fresh strawberry fruits. Statistical analysis criteria are as indicated in Figure 2.

The ascorbic acid content in the strawberry fruits was evaluated using HPTLC analysis. The results show that both NaturGel and PriobioHumus used alone or in combination significantly increased the ascorbic acid content in strawberry fruits (Figure 4). The highest content of vitamin C was found in fruits harvested from plants treated with ProbioHumus (53.75 mg 100 g^−1^ FW). The average ascorbic acid content in strawberry fruits after treatment with a mixture of ProbioHumus and NaturGel was 49.55 mg 100 g^−1^ FW, and after treatment with NaturGel, it was 35.75 mg 100 g^−1^ FW. In the case of ProbioHumus and a combination of the two probiotics, the contents of vitamin C were significantly different from the control group (27.23 mg 100 g^−1^ FW) (Kruskal–Wallis criterion, χ^2^ (1) = 18.389, *p* < 0.05).

#### 2.2.4. Antioxidant Activity

**Figure 5:**
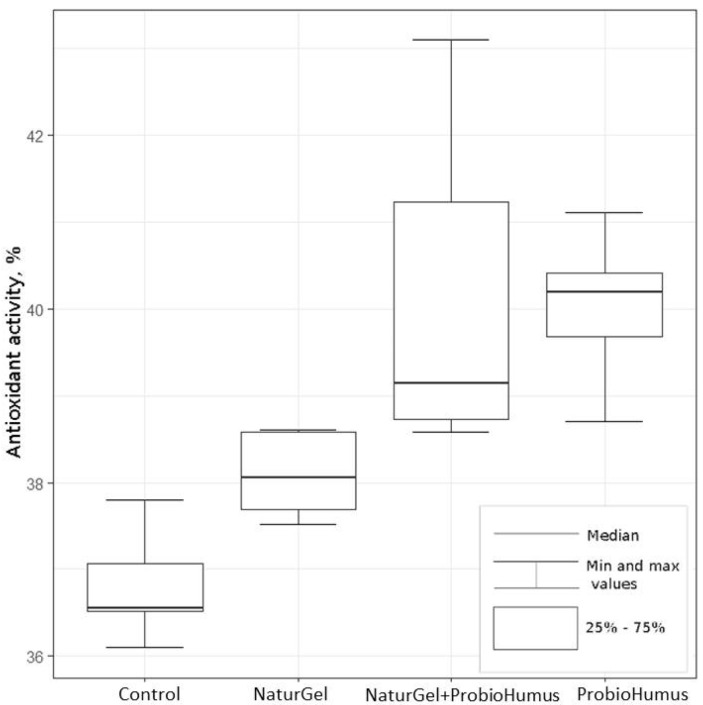
The impact of the probiotic preparations NaturGel and ProbioHumus on the antioxidant activity in fresh strawberry fruits. Statistical analysis criteria are as indicated in Figure 2.

The effect of the probiotic preparations NaturGel and ProbioHumus on the antioxidant activity of fresh strawberry fruits was estimated by the DPPH assay. The data show that the NaturGel and ProbioHumus preparations significantly increased the antioxidant activity in strawberries compared to controls (Figure 5). The highest level of antioxidant activity was detected in strawberry fruits from the plants treated with ProbioHumus, with a median of 40.07% (Kruskal–Wallis criterion, χ^2^ (1) = 11.29, *p* < 0.001). Significant differences were also observed between strawberry fruits treated with NaturGel, 38.05% (Kruskal–Wallis criterion, χ^2^ (1) = 9.27, *p* < 0.05), and NaturGel + ProbioHumus, 39.14% (Kruskal–Wallis criterion, χ^2^ (1) = 9.60, *p* < 0.05), that were statistically different from the non-treated control, 36.55% (Figure 5).

## 3. Discussion

Recently, consumers’ demand for high-quality organically grown functional food has increased significantly [25]. This is especially true for products such as berries, fruits, and vegetables, a large proportion of which are consumed fresh. Therefore, methods of promoting plant growth for sustainable agriculture are being studied worldwide [23]. It should be added that different cultivation systems also lead to different growth conditions and affect the phytochemical composition and antioxidant activity of strawberry fruits [8,25]. It is known that plant nutrition and secondary metabolic pathways can be affected by beneficial microorganisms [25,34,35,36,37]. It should be noted that the production of secondary metabolites is often low, less than 1% of the dry weight [38]. One of the most environmentally friendly biotechnological strategies to increase plant productivity is the use of microbial elicitors that improve biomass production by initiating defensive reactions while promoting secondary plant metabolism [37,39,40,41]. Several studies have confirmed that probiotic microorganisms can significantly improve not only the growth of crops but also the quality of food, by increasing the content of some bioactive compounds that are beneficial to human health [22]. The results of our study reveal an improvement in the growth and quality of strawberries grown on an organic farm when plants were treated with the probiotics ProbioHumus and NaturGel. Strawberry plants treated with both ProbioHumus and NaturGel preparations and their combination significantly increased fresh fruit weight compared to non-treated control plants (Table 1). The increase in the fresh weight of strawberry fruits may be due to an increase in dry matter accumulation. The highest dry and fresh weights were recorded for the fruits harvested from plants treated with the probiotic ProbioHumus. Similar results were obtained with blackcurrant berries: the fresh weight of berries harvested from plants treated with ProbioHumus increased by 45%, and the crop yield was even 1.1 t ha^−1^ higher than that of non-treated control plants [42]. These data are in agreement with the results of other studies on the effect of plant probiotic bacteria on strawberry growth and yield [20,23,31,32,43,44,45]. The weight of the tested strawberry fruits was lower than that of the same variety grown under intensive cultivation conditions but is consistent with other studies where strawberry crops were reduced under fertilization [31] and ecological conditions [20]. It is known that the reduction in yield due to smaller fruits is a serious problem for organic farms, which, as the results of this study have shown, can be successfully addressed by the use of plant probiotics. Improved growth of fruits of treated plants may be associated with increased plant nutrient use efficiency and/or endogenous changes in phytohormone levels and other mechanisms. There are a number of research studies proving that plant probiotic solubilization and mineralization of nutrient components, especially P minerals, perform biological N2 fixation, produce growth-stimulating phytohormones, prevent pathogen-induced plant diseases, and increase plant resistance to biotic or abiotic stresses [23,25,26,28,29,30].

Phenolic compounds as plant secondary metabolites are widespread in plants and have a large range of structures and functions. The health benefits of these phytochemicals are directly related to their antioxidant potential and the relationship between their use and the prevention of some diseases [2,4,46]. Strawberries are on the list of 100 foods and beverages richest in polyphenols [47]. Approximately 40 phenolic compounds with different structures and functions have been identified in strawberry fruits [2]. The content of identified polyphenols varies from 99.4 to 1441 mg GAE 100 g^−1^ FW [5,6,15,17,48,49]. In our study, the concentration of total phenolic compounds estimated in the fruits of control strawberry plants ranged from 313 to 438 mg GAE 100 g^−1^ FW. This corresponds to the total amounts of phenolic compounds reported by other researchers. The results show that the content of phenolic compounds in the strawberries treated with the tested probiotic preparations remained at the control level. Meanwhile, other authors, such as Rahman et al. [23], showed a significantly higher content of total phenols in the fruits of strawberries treated with *Bacillus amylolequifaciens* Bchi1 and *Paraburkholderia fungorum* BRRh-4 isolates compared to the non-treated control. A higher content of total phenols was also found by Ramos-Solano et al. [50] in blackberries inoculated with a *Pseudomonas* strain (N21.4) compared to non-inoculated blackberries. 

One of the most important characteristics in assessing the quality of strawberries is the attractive red color, caused by anthocyanins, a group of water-soluble phenolic compounds with antioxidant properties [2,5,51]. According to the data of multiple studies, the content of anthocyanins in strawberries ranges from 2.09 to 48 mg 100 g^−1^ FW [5,6,15,17,48,52] and is predetermined by the cultivar or maturing stage [5,53]. Their accumulation is strongly influenced by the cultivation system and environmental conditions such as the temperature, light intensity, and soil composition [4,15,54]. The results obtained in our study show that the content of anthocyanins in control strawberries ranged from 26.5 to 34.4 mg 100 g^−1^ FW and corresponds to the content of anthocyanins determined by other authors in various strawberry varieties. The probiotic preparations NaturGel and ProbioHumus did not increase the anthocyanin content in strawberry fruits. Meanwhile, Lingua et al. [25] showed that the anthocyanin concentration in strawberry fruits was increased in plants inoculated with a mixture of a mycorrhizal fungus (*Glomus* sp.) and plant growth-promoting bacterial strains (*Pseudomonas fluorescens Pf4* and *5Vm1K)* under conditions of reduced fertilization. Anthocyanin levels were also increased in strawberry fruits by the application of both *Bacillus amylolequifaciens* Bchi1 and *Paraburkholderia fungorum* BRRh-4 isolates [23]. In contrast, no changes in the anthocyanin content were observed in the study with blackberries inoculated with *Pseudomonas* [50]. The anthocyanin concentration depends on the counterbalance between its synthesis and degradation. Indeed, the decreased anthocyanin concentration cannot simply be explained, and no information is available on the influence of plant probiotics on the contents of anthocyanins of strawberry fruits. In blackcurrant berries, there was a moderate negative correlation between ascorbic acid and anthocyanins (R = −0.52, *p* < 0.00001) [42]. It is apparent that the increased antioxidant activity of strawberries from plants treated with probiotics correlated with strong antioxidants such as phenolic compounds, anthocyanins, and ascorbic acid. 

Ascorbic acid is vital for human health and disease prevention; in blood plasma, it has been shown to have the highest correlation with fruit and vegetable intake [8,55]. Ascorbic acid is considered to be one of the most important nutritional components on which the antioxidant potential of strawberry fruits also depends [3,32,55]. Szeto et al. [10] showed that strawberry fruits had the highest content of ascorbic acid among the 17 different fruits studied. It was higher than the content of ascorbic acid in kiwis and lemons. When compared to other berry species, the content of ascorbate in strawberries is similar to that of raspberries, but about 4-fold higher than that of blueberries [4]. Different authors reported various amounts of ascorbate (from 5 to 104 mg 100 g^−1^ FW) in strawberry fruits [4,10,14,17]. Similar ranges of the ascorbic acid content (from 14.2 to 79.74 mg100 g^−1^ FW) were found in the current study. Our results show that all treatments with probiotics caused a significant increase in the ascorbic acid content compared to the control (Figure 4). The highest levels of ascorbic acid (31.4–72.5 mg 100 g^−1^ FW) were found in strawberry fruits treated with ProbioHumus, where its concentrations almost doubled compared to control plants (14.2–40.9 mg 100 g^−1^ FW). A significant increase in the ascorbic acid content was also observed in strawberry fruits treated with NaturGel + ProbioHumus (27.5–79.7 mg 100 g^−1^ FW) and NaturGel (18.7–49.7 mg 100 g^−1^ FW), with increases of 82% and 31% compared to the control, respectively. Contradictory data have been found in the literature regarding changes in the ascorbic acid content following treatment of strawberry plants with plant growth-promoting probiotics. No changes in the vitamin C concentration were found by Pırlak and Köse [43] in strawberries inoculated with *Pseudomonas* BA-8, *Bacillus* OSU-142, and *Bacillus* M-3, and by Esikten et al. [20] in plants inoculated with *Pseudomonas* and *Bacillus* strains. Meanwhile, Erturk et al. [44] showed an increase in the vitamin C content in strawberries inoculated with *Paenibacillus polymyxa* RC05. Flores-Félix et al. [32] inoculated strawberry plants with *Phyllobacterium endophyticum* under greenhouse conditions and obtained an almost 2-fold higher vitamin C content in strawberry fruits than in fruits of non-inoculated plants. Ascorbic acid production in strawberry fruits was also increased by inoculation with a mixture of arbuscular mycorrhizal fungi and two plant growth-promoting bacterial strains under reduced chemical fertilization conditions [31].

As mentioned before, the antioxidant activity of strawberries is high when compared to other vegetables and fruits [4,7,9,10,11]. The antioxidant activity of strawberries varies from 9.6% to 297% [6,15,49,56] and can fluctuate for many reasons [4]. The differences can be attributed to the genotype, degree of fruit ripening [6,49], and environmental factors such as climatic conditions, biotic interactions, light, irrigation, fertilization, and cultivation methods [14,16,25,54]. The results of our study show that the use of plant probiotic microorganisms has a definite significance for the antioxidant activity of strawberry fruits. Both probiotic preparations significantly increased the antioxidant activity of strawberry fruits in the case of the separate or combined treatment (Figure 5). The highest mean values were found in fruits of plants treated with ProbioHumus—the average median of inhibition in the DPPH assay was 40.07%, and in strawberries treated with NaturGel + ProbioHumus, it was 39.14%. The highest antioxidant abilities (up to 43.1%) were recorded in the fruits of strawberries treated with a mixture of both probiotics. Significantly higher antioxidant activity was detected in NaturGel-treated strawberries, 38.05% (Kruskal–Wallis criterion, χ^2^ (1) = 9.27, *p* < 0.05), when compared to control strawberries (36.55%). These results are consistent with data from other authors. Rahman et al. [23] showed that the total antioxidant activity of field strawberry plants treated with the probiotic bacteria *B. amyloliquefaciens* and *Paraburkholderia fungorum* was significantly higher than that of non-treated control plants. It is evident that the microbial treatments initiate plant defense reactions while stimulating secondary plant metabolism and antioxidant production.

Several studies have been performed to determine which antioxidant compounds have the greatest effect on the overall antioxidant activity of strawberries. Aaby et al. [12] found the highest contribution of ascorbic acid (24%) and ellagitannins (19%), and the third position in terms of antioxidant capacity was assigned to anthocyanins (13%). Tulipani et al. [13] reported the same trend, with ascorbic acid accounting for more than 30% and anthocyanins for 25–40% of the total strawberry antioxidant activity, with ellagitannin derivatives and flavanols accounting for the rest. The moderate correlation between the antioxidant activity and ascorbic acid content (R = 0.51694 at *p* < 0.05) obtained in our study coincides with the data of other authors [14] and confirms that the antioxidant activity of strawberries was significantly affected by the vitamin C content.

In this study, we showed that treatment of strawberry plants with the ProbioHumus and NaturGel probiotics and their mixture increased not only fruit growth but also the content of antioxidants, especially vitamin C, and significantly increased fruit antioxidant activity when compared to control plants. Thus, treatment with the probiotics ProbioHumus and NaturGel enabled the cultivation of high-quality and functional strawberry fruits under organic farming conditions. This is beneficial for both agriculture and the environment, as well as for human health, and thus the studied microbial probiotics can be used as biofertilizers for the production of functional fruits in sustainable organic farming systems.

## 4. Materials and Methods

### 4.1. Plant Material

Strawberry (*Fragaria × ananassa*) cv. ‘Deluxe’ plants were grown in organic farm fields in Prienai District, Lithuania, 56°05′ N 24°41′ E, on typical saturated common Arenosols. The main agrochemical parameters of the arable soil layer of the farm were pH 6.8–7.1, N_min_ 14.38–18.95 mg kg^−1^, P_2_O_5_ 459.2–505.6 mg kg^−1^, and K_2_O 125.3–214.1 mg kg^−1^. Strawberry plants were planted in April–May 2018, planting E class frigo seedlings in matte rows, about 36,000 plants per hectare. Fruits were collected for sampling on 25 June 2019.

For each variant, fruit samples of a similar size and uniform maturity, as determined by the surface color, were collected randomly from 40 different plants at different locations in the field. The freshly sampled fruits were measured and weighed. From the collected fruit mass of each variant, 5 kg of fruit was selected by random sampling to avoid uncertainty due to sample inhomogeneity and homogenized. Then, one part of the homogenate was immediately frozen and stored at −80 °C until biochemical analysis. The other part was used to estimate the dry fruit mass. An amount of 5 g of the fruit homogenate was dried at 60 °C for 7 days until dry and weighed. Then, the percentage of water content was calculated [31].

### 4.2. Probiotic Preparations ProbioHumus and NaturGel

The commercial probiotic preparation ProbioHumus (purchased directly from the manufacturer) is a dark brown liquid with a sweet smell of fermented yeast, pH 3.5 ± 0.3. It is composed of microorganisms: *Bacillus subtilis*; yeast *Saccharomyces cerevisiae*; lactic acid bacteria *Bifidobacterium animalis*, *B. bifidum*, *B. longum*, *Lactobacillus diacetylactis*, *L. casei*, *L. delbrueckii*, *L. plantarum*, *Lactococcus lactis*, *Streptococcus thermophilus*; phototropic bacteria *Rhodopseudomonas palustris*, *R. sphaeroides* (purchased from Baltic Probiotics, South Kurzeme region, Latvia). All ingredients used in the production of ProbioHumus are 100% of natural origin and standard, not derived from genetically modified materials.

The commercial organic fertilizer and probiotic preparation produced by HydroThermoDynamic Technology, NaturGel (purchased directly from the manufacturer), contains enzymes, amino acids, vitamins (B1, B2, PP, E, A, *carotenoids*), fulvic and humic acids, carbohydrates, *Azotobacter*, *Bacillus*, *Rhizobium*, *Bradyrhizobium*, *Lactobacillus*, and *Trichoderma* microorganism genera, macroelements: nitrogen 30 g/L, phosphorus 3.1 g/L, potassium 0.5 g/L, and microelements: magnesium 100 mg/L, iron 100 mg/L, manganese 13.3 mg/L, zinc 8.0 mg/L, copper 1.0 mg/L, cobalt 0.7 mg/L, boron 0.5 mg/L, molibden 0.2 mg/L (purchased from Sadera, Vilnius, Lithuania).

### 4.3. Plant Treatment with Probiotic Preparations

**Figure 6:**
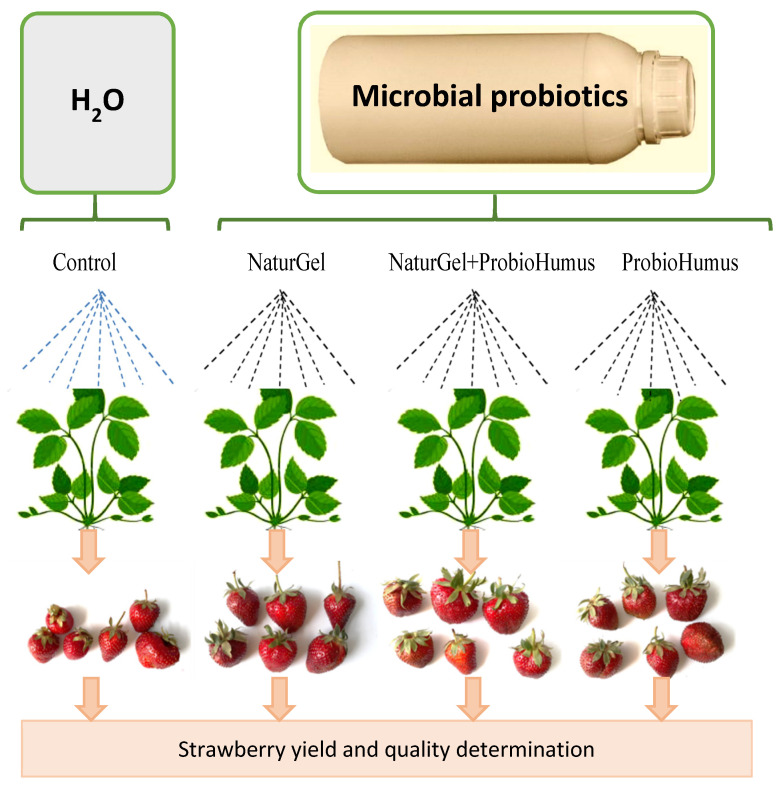
The experimental setup.

Four experimental plots (4 × 1 hectare) were arranged in order to test the effect of ProbioHumus, NaturGel, and their combined effect (Figure 6). Plants were treated with the tested probiotic preparations twice a year: at the beginning of dormancy after harvest BBCH 91–92 (in the fall of 2018) and at the leaf development (leaves spreading before flowering) BBCH 13–15 stage (in the spring of 2019) [57]. The probiotics were diluted 1:100 with water and sprayed onto the plants (4 L probiotic concentrate ha^−1^) according to the scheme. In the combined treatment, equal volumes of ProbioHumus and NaturGel were diluted 1:100 with water and sprayed onto the plants (4 L probiotic concentrate ha^−1^). The control field was sprayed with water.

### 4.4. Extraction

For evaluation of the content of bioactive compounds, the extracts were prepared by homogenizing the berries with a medium at a 1:10 ratio of plant material in a porcelain mortar and pestle. The medium consisted of 90% aqueous methanol (Rotisolv, Roth, Karlsruhe, Germany) acidified with 0.1 N hydrochloric acid (Fluka Analytical, Munich, Germany). The homogenate was stirred with a magnetic stirrer for 30 min. The sealed homogenates were stirred with a magnetic stirrer (MM 2A, Czech) for 16 h in the dark at 4 °C, and the precipitate was then removed using a water vacuum pump with a 0.22 μm membrane filter (Millipore, Darmstadt, Germany).

The berries were homogenized in 50% methanol for ascorbic acid analysis. The homogenate was centrifuged for 15 min at 4 °C at a speed of 3000 rpm (centrifuge MPW-351 R, MPW Med. instruments, Warsaw, Poland). Prior to analysis, the extracts were stored for 16 h at 4 °C in the dark.

### 4.5. Antioxidant Activity Determination

The antioxidant activity of strawberry extracts was measured using the DPPH free radical scavenging method [58]. A methanol solution (Rotisolv HPLC) of 6.5 × 10^−5^ M DPPH (Fluka) was stirred for 3 h at 4 °C in the dark. Strawberry fruit extracts were diluted 1:20 with DPPH solution and incubated for 30 min at 25 °C in the dark. For the blank sample, methanol was used instead of berry extracts. The decrease in DPPH absorbance was measured spectrophotometrically (Analytik Jena Specord 210 plus, Jena, Germany) at 515 nm. BHT was used as a reference (Sigma-Aldrich, St. Louis, MO, USA), and DPPH radical scavenging activity (%) was calculated as (Ac − As) × 100/Ac, where Ac is the absorbance of the blank sample, and As is the absorbance of the sample.

### 4.6. Determination of Total Phenols

The total phenolic content of the strawberry extracts was determined according to the Folin–Ciocalteu colorimetric protocol from Singleton et al. [59]. The fruit extracts were mixed with a 10-fold dilution of Folin–Ciocalteu reagent (Sigma-Aldrich, Burlington, MA, USA) and a solution of 7.5% sodium carbonate (Sigma-Aldrich, Burlington, MA, USA) at a ratio of 1:5:4 *v*/*v*/*v*. A blank sample was prepared with acidified methanol instead of the fruit extracts. Samples were incubated for 30 min at ambient temperature in the dark. The absorbance of samples was measured at 765 nm. A blank sample was used as a reference. 

The total phenolic content was calculated as gallic acid equivalent mg g^−1^ of fresh weight (FW) (R^2^ = 0.99). 

### 4.7. Determination of Anthocyanin Content

The total anthocyanins were determined using the pH difference method as reported by Giusti and Wrolstad [60]. A mixture of strawberry extract with 0.025 M potassium chloride buffer (Sigma-Aldrich, Burlington, MA, USA) (pH 1.0) and 0,4 M sodium acetate buffer (Sigma-Aldrich, Burlington, MA, USA) (pH 4.5) at a ratio of 1:4 *v*/*v* was incubated in the dark for 30 min. Measurement of absorbance was carried out using a spectrophotometer at 520 nm and 700 nm. The values were reported as mg cyanidin-3-glucoside equivalents per 100 g^−1^ FW.

### 4.8. Ascorbic Acid Determination

The content of ascorbic acid was determined according to the HPTLC method reported by Chakraborthy [61] with minor modifications. Standard solutions of ascorbic acid (Sigma-Aldrich, Burlington, MA, USA) were prepared by dissolving them in absolute ethanol (Stumbras, Vilnius, Lithuania). The standard solution and berry extracts were applied as 6 mm bands using a TLC 4 automated sampler (Camag, Muttenz, Switzerland) by spraying with a 25 µL dosing syringe (Hamilton, OH, USA) to a 20 cm × 10 cm glass silica gel chromatography plate. The automatic developing chamber ADC 2 provided relative humidity conditions of 33%. The mixture of ethanol (96%)/glacial acetic acid (Carl Roth GmbH + Co KG) with the rate of 9.5:0.5 (*v*/*v*) was used as the mobile phase. Following separation, the air-dried plate was scanned at 256 nm using a TLC 2 visualizer and a TLC 4 densitometer. The measurements were conducted using a TLC Scanner 3 operated in the absorbance mode and controlled by the winCATS 1.4.2 software. The obtained data were derived from the results of sample peaks, analogous to ascorbic acid in the calibration curve.

### 4.9. Statistical Analysis

The statistical analysis was conducted using Rstudio (USA). The Kruskal–Wallis test was used to assess the effect of the probiotic preparations tested. The correlation between the groups was determined using Spearman’s ρ coefficient. Significant differences were considered to be those with *p*-values < 0.05. All morphometric and performance data were statistically analyzed using one-way ANOVA. Tukey’s test was used to analyze the statistical significance of differences (*p* < 0.05) between means. Values presented are means ± standard deviations (SD) of at least three experiments, each involving five replicates.

## 5. Conclusions

1. ProbioHumus and NaturGel, used separately and in combination, significantly increased the biomass of organic field strawberries by 42.4%, 35.6%, and 37.3%, respectively.

2. The fruits of plants treated with probiotics contained a higher amount of antioxidants, especially ascorbic acid. The ascorbic acid content almost doubled compared to the non-treated strawberry fruits.

3. Strawberry fruits harvested from plants treated with microbial probiotics showed a significantly (*p* < 0.05) higher ability to scavenge DPPH free radicals.

4. The effect of commercial plant probiotics on strawberry production was investigated under field conditions on organic farms. The products were effective at very low doses and can therefore be recommended as a cultivation element for the development of environmentally friendly, high-quality strawberry cultivation techniques with little or no additional use of inorganic fertilizers.

## Figures and Tables

**Table 1:** The impact of the probiotic preparations NaturGel and ProbioHumus on the biometric parameters of strawberry fruits.

Parameters	Control	NaturGel	NaturGel + ProbioHumus	ProbioHumus
Fresh weight, g	5.9 ± 0.28	8.0 ± 0.51 a	8.1 ± 0.39 a	8.4 ± 0.50 a
Length, cm	2.18 ± 0.03 a	2.54 ± 0.05 b	2.42 ± 0.04 ab	2.28 ± 0.03 ab
Diameter, cm	2.31 ± 0.02 a	2.56 ± 0.03 b	2.60 ± 0.05 b	2.45 ± 0.04 ab
Dry biomass, %	12.76 ± 0.50	13.74 ± 0.61 ab	13.44 ± 0.59 b	14.09 ± 0.74 a

Mean values followed by the same letters are not significantly different at *p* ≤ 0.05.

## Data Availability

The data supporting the reported results can be found in the archive of scientific reports of the Nature Research Centre.
